# Classifying the tracing difficulty of 3D neuron image blocks based on deep learning

**DOI:** 10.1186/s40708-021-00146-0

**Published:** 2021-11-05

**Authors:** Bin Yang, Jiajin Huang, Gaowei Wu, Jian Yang

**Affiliations:** 1 Faculty of Information Technology, Beijing University of Technology, Beijing, China; 2 Beijing International Collaboration Base on Brain Informatics and Wisdom Services, Beijing, China; 3 School of Artificial Intelligence, University of Chinese Academy of Sciences, Beijing, China; 4 Institute of Automation, Chinese Academy of Sciences, Beijing, China

**Keywords:** Deep learning, Tracing difficulty classification, Residual neural network, Fully connected neural network, Long short-term memory network

## Abstract

Quickly and accurately tracing neuronal morphologies in large-scale volumetric microscopy data is a very challenging task. Most automatic algorithms for tracing multi-neuron in a whole brain are designed under the Ultra-Tracer framework, which begins the tracing of a neuron from its soma and traces all signals via a block-by-block strategy. Some neuron image blocks are easy for tracing and their automatic reconstructions are very accurate, and some others are difficult and their automatic reconstructions are inaccurate or incomplete. The former are called low Tracing Difficulty Blocks (low-TDBs), while the latter are called high Tracing Difficulty Blocks (high-TDBs). We design a model named 3D-SSM to classify the tracing difficulty of 3D neuron image blocks, which is based on 3D Residual neural Network (3D-ResNet), Fully Connected Neural Network (FCNN) and Long Short-Term Memory network (LSTM). 3D-SSM contains three modules: Structure Feature Extraction (SFE), Sequence Information Extraction (SIE) and Model Fusion (MF). SFE utilizes a 3D-ResNet and a FCNN to extract two kinds of features in 3D image blocks and their corresponding automatic reconstruction blocks. SIE uses two LSTMs to learn sequence information hidden in 3D image blocks. MF adopts a concatenation operation and a FCNN to combine outputs from SIE. 3D-SSM can be used as a stop condition of an automatic tracing algorithm in the Ultra-Tracer framework. With its help, neuronal signals in low-TDBs can be traced by the automatic algorithm and in high-TDBs may be reconstructed by annotators. 12732 training samples and 5342 test samples are constructed on neuron images of a whole mouse brain. The 3D-SSM achieves classification accuracy rates 87.04% on the training set and 84.07% on the test set. Furthermore, the trained 3D-SSM is tested on samples from another whole mouse brain and its accuracy rate is 83.21%.

## Introduction

Tracing morphologies of neurons is essential for investigating the structure and the function of neurons, exploring the working mechanism of the brain and studying the mechanism of brain diseases, such as neuron classification [1], neuron morphology analysis [2] and potential connectivity between brain circuits [3]. For small or medium scale volumetric light microscopy datasets, the BigNeuron project collected more than 30 automatic algorithms and transplanted them to the Vaa3D platform [4, 5], which can visualize 3D neuron images, produce automatic reconstructions and analyze neuronal morphologies. For large-scale image datasets [6, 7], Bria et al. developed a Vaa3D-Terafly open source tool to visualize, analyze and manage them [8], and Peng et al. proposed an Ultra-Tracer framework to trace their signals [9]. However, it is still a very challenging task to quickly and accurately trace neuronal morphologies in large-scale multi-neuron images of a whole mouse brain.

In the past decade, deep learning has achieved great success on many computer vision tasks, such as image classification [10–12], image segmentation [13–15], target detection [16–18], etc. Convolutional Neural Network (CNN) plays an important role in the field of image classification. Simonyan et al. proposed a VGG network [10] and it generated good results on ImageNet dataset [19] by using a small receptive field and more layers. Szegedy et al. designed an Inception network [11, 20], which uses convolution kernels with different sizes to increase the diversity of features and adopts a large number of $1 \times 1$ convolution kernels to reduce the number of network parameters. He et al. composed a Residual neural Network (ResNet) for image recognition, which builds a deeper neural network by utilizing skip connections to jump over some layers [12].

For automatic neuron tracing on large-scale multi-neuron images, deep learning has been used to solve many image related problems. Zhou et al. developed a DeepNeuron toolbox, which adopts deep neural networks to learn features and rules hidden in light microscopy images and traces neuronal morphologies [21]. Chen et al. presented a spherical-patches extraction and 2D multi-stream CNN based method, which can simultaneously detect all 3 types of 3D critical points in neuron microscopy images [22]. Liu et al. designed a deep learning based segmentation method to identify the location of neuronal voxels, which is capable of both enhancing neuronal signals and reducing image noise [23]. Jiang et al. proposed a method based on a ray-shooting model and a Long Short-Term Memory network (LSTM) [24], which is able to enhance weak-signal neuronal structures and remove background noise in 3D neuron images [25]. Although the above deep learning based methods have made some progresses on the multi-neuron reconstruction task, it is still very challenging to accurately and quickly trace neuronal morphologies in multi-neuron images of a whole mouse brain.

**Fig. 1:**
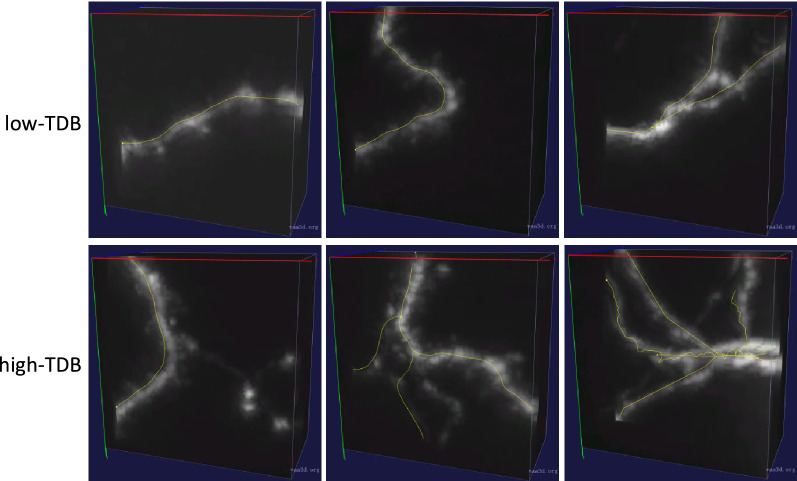
Examples of low-TDBs and high-TDBs

In 3D multi-neuron images of a whole mouse brain, there are some image blocks with simple morphology structures, strong signals and weak noises, on which many automatic algorithms can trace morphologies quite accurately. These image blocks (the first row in Fig. 1) are called low Tracing Difficulty Blocks (low-TDBs). While there are many other image blocks with complex morphology structures (including some bifurcations, crossing signals, etc.), weak signals or strong noises, on which most automatic algorithms trace morphologies inaccurately or incompletely. These image blocks (the second row in Fig. 1) are called high Tracing Difficulty Blocks (high-TDBs). If we can classify 3D image blocks as low-TDBs or high-TDBs, neuronal signals in low-TDBs can be traced by an automatic algorithm and in high-TDBs may be reconstructed by annotators. This interacting strategy of automatic tracing and manual annotating is capable of promoting the Ultra-Tracer framework to generate more accurate neuron reconstructions with higher efficiency. Therefore, it is interesting to study the tracing difficulty classification of 3D neuron image blocks.

In this paper, a model called 3D-SSM is designed to classify the tracing difficulty of 3D image blocks, which is based on ResNet [12], Fully Connected Neural Network (FCNN) [26] and Long Short-Term Memory network (LSTM) [24]. 3D-SSM consists of three modules: Structure Feature Extraction (***S***FE), Sequence Information Extraction (***S***IE) and Model Fusion (***M***F). SFE utilizes a 3D-ResNet and a FCNN to extract two kinds of features in 3D neuron image blocks and automatic reconstruction blocks. SIE uses two LSTMs to learn sequence information hidden in features of sequential blocks generated in SFE. MF adopts a concatenation operation and a FCNN to fuse outputs from SIE.

**Fig. 2:**
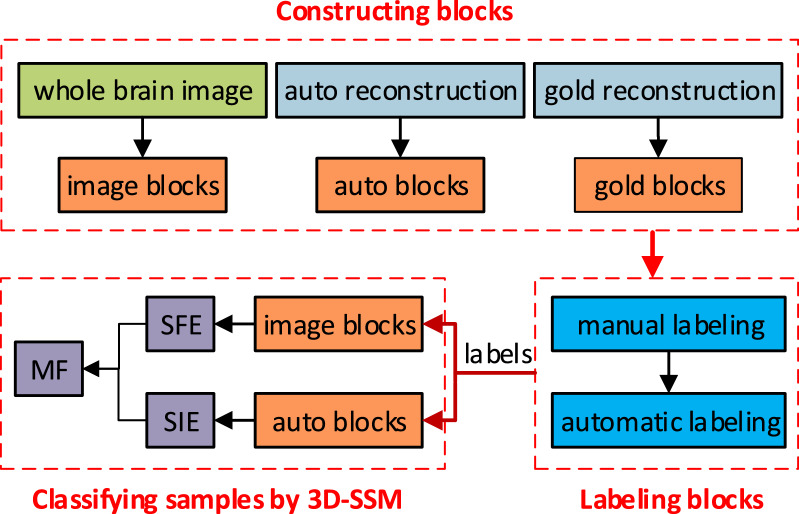
The framework of classifying the tracing difficulty of 3D image blocks

As shown in Fig. 2, we divide the implementation of classifying the tracing difficulty of 3D image blocks into three steps: constructing blocks, labeling blocks and classifying samples by 3D-SSM. Firstly, neuron images of a whole mouse brain are partitioned into many overlapped 3D image blocks along the gold standard reconstruction (reconstructed by professional annotators) of each neuron. Gold standard reconstruction blocks (gold blocks) and automatic reconstruction blocks (auto blocks) are parts of the gold standard reconstruction and automatic reconstruction located in each 3D image block, respectively. Then, each 3D image block is labeled as low-TDB or high-TDB by manual or an automatic algorithm. Finally, 3D image blocks and L-Measure [27] features of corresponding reconstructions are used to train and test 3D-SSM. It achieved classification accuracy rates of 87.04% and 84.07% on training set and test set, respectively. Results of the tracing difficulty classification produced by the 3D-SSM model can be used as a stop condition of an automatic tracing algorithm in the Ultra-Tracer framework. With that, neuronal signals in low-TDBs can be traced by the automatic algorithm and in high-TDBs may be reconstructed by annotators. The interaction between automatic tracing and manual reconstruction is capable of promoting the Ultra-Tracer framework to generate more accurate neuron reconstructions efficiently.

This paper has the following three contributions:

- The task on classifying the tracing difficulty of 3D neuron image blocks is proposed, and its solution procedure is designed.
- More than 23000 samples are constructed on two whole mouse brains, and manual labeling and automatic labeling are used to generate the label of these samples.
- Based on 3D-ResNet, FCNN and LSTM, a 3D-SSM model is proposed to classify the tracing difficulty of 3D image blocks, and it has good performance on 3D image blocks of two whole mouse brains.

The rest of the paper is organized as follows. In Section 2, we introduce how to construct sample data including producing block data, extracting features and labeling samples. In Section 3, we present the 3D-SSM model including its three modules: SFE, SIE and MF. The experimental results are reported in Section 4, and conclusions and discussions are in Section 5.

## Sample data

Sample data is constructed from 3D neuron images of two whole mouse brains (denoted by brain-A and brain-B, respectively), gold standard reconstructions and automatic reconstructions of marked neurons in them. These data were provided by the Southeast University-Allen Institute Joint Center. For each 3D image block, its corresponding gold standard reconstruction blocks (gold blocks) and automatic reconstruction blocks (auto blocks) are taken out for extracting features. Neuron distance [28] features between gold blocks and auto blocks are used to describe their similarity, and L-Measure [27] features of auto blocks are employed to characterize the neuronal structures in 3D image blocks. The label of a 3D image block is its tracing difficulty, low-TDB or high-TDB.

### Constructing blocks

Gold standard reconstructions of neurons in brain-A and brain-B were drawn and checked by at least three professional annotators using the Vaa3D-Terafly visualization software [8], and are stored in SWC format [29] which describes the morphology of a neuron as tree structures with location, node’s radius, parent node and some other attributes. There are 93 and 37 gold standard reconstructions of marked neurons in brain-A and brain-B respectively. Automatic reconstructions are generated by the APP2 algorithm [30] under the Ultra-Tracer framework, which is one of the state-of-the-art automatic tracing methods and can efficiently produce good reconstructions for many complex neurons.

**Fig. 3:**
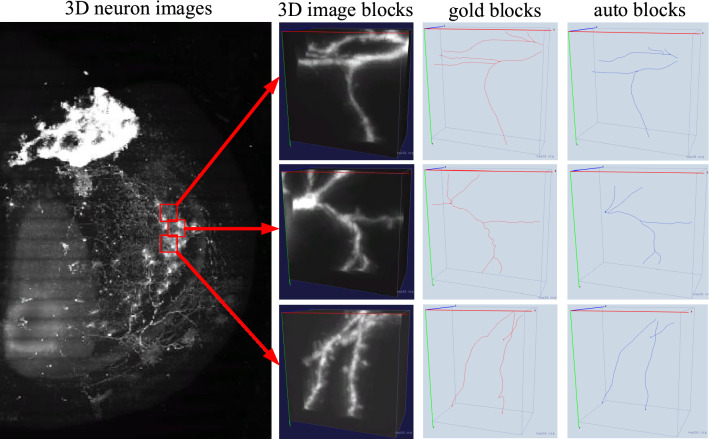
Constructing 3D image blocks, gold blocks and auto blocks from 3D neuron images, gold standard reconstructions and automatic reconstructions, respectively

From the root node of each neuron (corresponding to its soma) and moving along its gold standard reconstruction with step 100 nodes, 3D neuron images are partitioned into many overlapped 3D image blocks with size $32 \times 64$ $\times$64. Gold blocks and auto blocks are parts of the gold standard reconstruction and APP2 reconstruction respectively, which are located in corresponding 3D image blocks. As illustrated in Fig. 3, three nodes with distance 100 nodes in a gold standard reconstruction are selected as centers (anchors) of three 3D image blocks (the second column in Fig. 3), and their corresponding gold blocks (the third column in Fig. 3) and auto blocks (the forth column in Fig. 3) are obtained.

There are some burrs in auto blocks which may be noises and meaningless. We delete branches with nodes less than 4, and fragments with nodes less than 4 and distance to the edge of the image block less than 4 voxels. There are some auto blocks with too few reconstruction nodes, which means that few neuronal signals in these blocks and inadequate information to evaluate the quality of reconstructions. 3D image blocks corresponding to auto blocks with total nodes less than 11 are filtered out. Finally, 18074 samples (3D image blocks) from brain-A and 5109 samples from brain-B are obtained, and their corresponding gold blocks and auto blocks are also taken out from gold standard reconstructions and APP2 reconstructions.

### Extracting reconstructions based features

The Entire Structure Average (ESA), Different Structure Average (DSA) and Percentage of Different Structures (PDS) are often used to measure the similarity between two reconstructions [28]. The following 7 distances are taken as neuron distance features: $\hbox {ESA}_{{12}}$ (the average distance of all nodes in reconstruction 1 to nodes in reconstruction 2), $\hbox {ESA}_{{21}}$ (the average distance of all nodes in reconstruction 2 to nodes in reconstruction 1), ESA (the average of $\hbox {ESA}_{{12}}$ and $\hbox {ESA}_{{21}}$), $\hbox {DSA}_{{2}}$ (the average distance of nodes with distance larger than two voxels), $\hbox {PDS}_{{12}}$ (the percentage of nodes in reconstruction 1 that have distance no less than two pixels to nodes in reconstruction 2) and $\hbox {PDS}_{{21}}$ (the percentage of nodes in reconstruction 2 that have distance no less than two pixels to nodes in reconstruction 1) and PDS (the percentage of nodes in reconstruction 1 or reconstruction 2 that have distance no less than two pixels to nodes in the other reconstruction). The smaller these values are, the more similar two reconstructions are. Above 7 neuron distance features for gold blocks and auto blocks can be obtained by calling the neuron distance plug-in in the Vaa3D platform. In addition, 3 morphology features: the number of bifurcations, the number of nodes and the number of fragments, are also used to describe the similarity between a pair of a gold block and an auto block.

L-Measure developed by Scorcioni et al. [27], is a toolkit for extracting neuronal morphology features (http://cng.gmu.edu:8080/Lm/help/index.htm). There are 43 morphology features of neuron fragments, such as length, width, height, angle, etc. We select 32 L-Measure features to describe auto blocks and other 11 L-Measures features do not make sense for neuron fragments in auto blocks.

### Labeling samples

Labeling samples is an important step in supervised classification tasks. Gold blocks and auto blocks are used to generate labels of 3D image blocks. For an automatic tracing algorithm, the tracing difficulty (low or high) of a 3D image block can be determined according to the similarity between its corresponding gold block and auto block. If they are very consistent, the 3D image block is labeled as low tracing difficulty (low-TDB), otherwise as high tracing difficulty (high-TDB). Since tree structures in gold blocks and auto blocks are very complex and diverse, it is quite difficult to accurately quantify their consistency. Each pair of gold block and auto block are visualized on the Vaa3D platform, and carefully compared by our annotators. After observing and comparing lots of pairs, 4 category rules are induced to label a 3D image block as low-TDB or high-TDB (https://github.com/BingooYang/Tracing-difficulty-classification-on-3D-neuron-image-block). According to these rules, 2954 3D image blocks from brain-A are labeled by one annotator and checked by other two annotators. However, manual labeling is very time consuming and automatic labeling has to be adopted. Extracted 7 neuron distance features and 3 morphology features of gold blocks and auto blocks are used to describe the similarity between each pair of gold block and auto block. If the similarity of a pair is very high, the automatic tracing algorithm performs quite well on the corresponding 3D image block and it is labeled as low-TDB, otherwise it is labeled as high-TDB. Above 2954 manually labeled pairs are used to train and test a FCNN model to classify the similarity of all gold block and auto block pairs. The FCNN model consists of four linear layers, and the number of nodes from the first layer to the fourth layer is 50, 30, 20 and 2, respectively. It produces accuracy rates of 96.9% and 96.4% on the training set and test set, respectively. So the trained FCNN has good performance and can be used to generate the label of remaining 3D image blocks. It is worth noting that this model utilizes gold standard reconstructions to learn the label of 3D image blocks. But more often, we don’t have a gold standard reconstruction and the trained FCNN can not solve the tracing difficulty classification task on 3D image blocks.

**Fig. 4:**
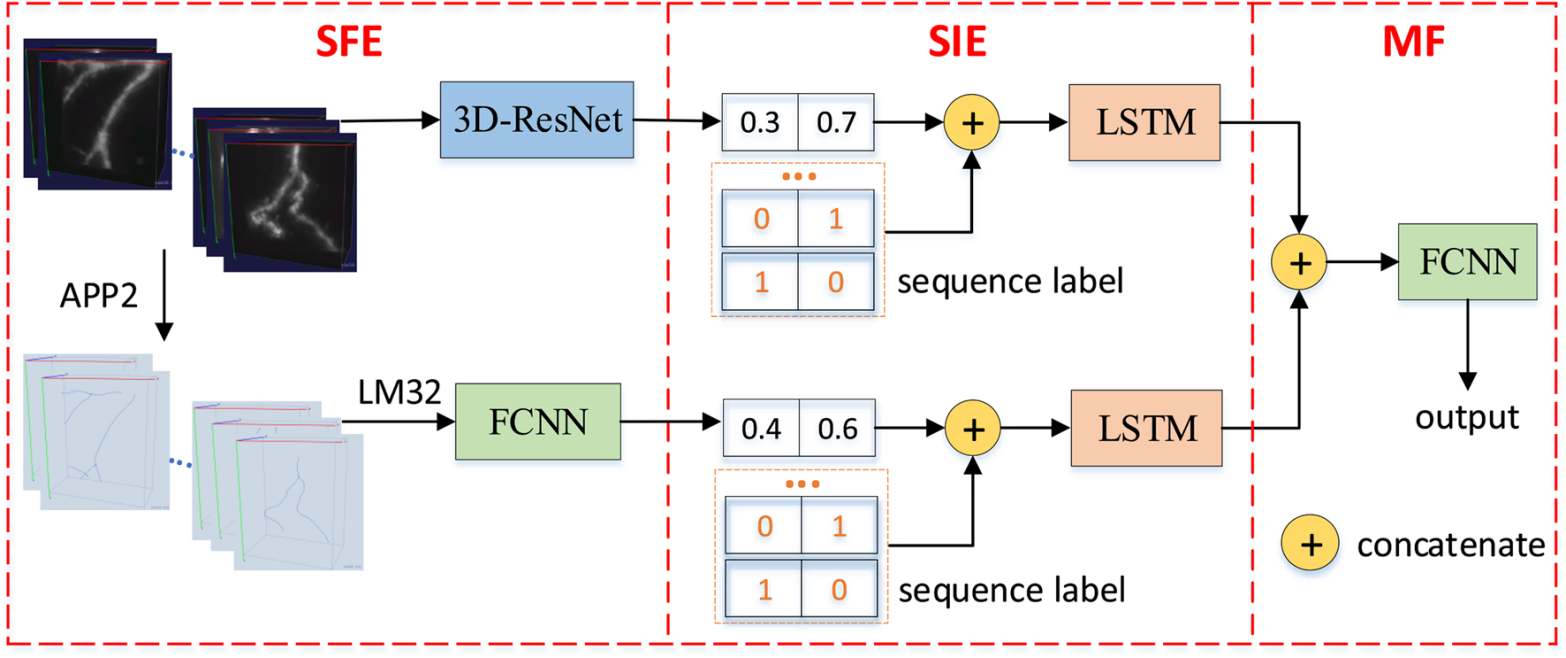
The structure of the proposed 3D-SSM model

## Method

A deep learning based 3D-SSM model is designed to classify the tracing difficulty of 3D image blocks. As illustrated in Fig. 4, 3D-SSM consists of three modules: Structure Feature Extraction (***S***FE), Sequence Information Extraction (***S***IE) and Model Fusion (***M***F). In SFE, a 3D-ResNet and a FCNN are trained by taking 3D image blocks and 32 L-Measure features of auto blocks as inputs, and their parameters are saved. In SIE, two LSTMs are adopted to extract the sequence information hidden in 3D image blocks and auto blocks, and the network parameters are also saved. In MF, the outputs of 3D image blocks and auto blocks produced in SIE are concatenated and taken as the inputs of a FCNN, and SFE, SIE and the FCNN are trained together.

**Table 1:** The structure of 3D-ResNet

Stage	Component	Output size
Convolution	$3\times 3\times 3$, 64, stride(1,2,2)	$32\times 32\times 32$
Max pooling	$3\times 3\times 3$, 64, stride(2,2,2)	$16\times 16\times 16$
Residual layer 1	Dropout=0.2, unit-A(64), unit-A(64)	$16\times 16\times 16$
Residual layer 2	Dropout=0.2, unit-B(128), unit-A(128)	$8\times 8\times 8$
Residual layer 3	Dropout=0.2, unit-B(256), unit-A(256)	$4\times 4\times 4$
Residual layer 4	Dropout=0.2, unit-B(512), unit-A(512)	$2\times 2\times 2$
Average pooling	$2\times 2\times 2$, stride(2,2,2)	$1\times 1\times 1$
Classification layer	Fully-connected, softmax	2

* Unit-A(*n*) consists of a 3$\times$3$\times$3 convolution with a 1$\times$1$\times$1 stride and *n* channels, a batch normalization, and an activation function (ReLU). Unit-B(*n*) has the same structure as unit-A(*n*) but a different convolution step size 2$\times$2$\times$2

### SFE of 3D-SSM

SFE contains two networks, a 3D image block based 3D-ResNet and an auto block based FCNN, which are used to extract structure features of 3D image blocks and auto blocks, respectively. The 3D image block based 3D-ResNet is to label a 3D image block as low-TDB or high-TDB by using feature maps of 3D images. The 3D-ResNet is designed on a usual ResNet, and contains convolution, pooling, batch normalization, dropout, skip connections and so on. Its inputs are 3D image blocks, output is a two-dimensional feature vector and its network structure is given in Table 1. The auto block based FCNN is to label a 3D image block as low-TDB or high-TDB by using features of auto blocks. That is to say, the tracing difficulty of a 3D image block is evaluated only by morphology features of its corresponding auto blocks. The FCNN is composed of three linear layers, and the number of nodes from the first layer to the third layer is 100, 50 and 2, respectively. Its inputs are extracted 32 L-Measure features and output is a two-dimensional feature vector.

It should be noted that outputs of the 3D-ResNet and the FCNN are set as 2 dimensional vectors for the following two purposes. The first one is to calculate their cross entropy loss with the one-hot encoding labels and so as to train the 3D-ResNet and the FCNN separately. The second one is to concatenate them with one-hot encoding sequence labels of 3D image blocks in the SIE module.

### SIE of 3D-SSM

SIE consists of two LSTMs for extracting sequence information hidden in 3D image blocks. Both of them have two layers and each layer consists of 10 hidden nodes. The sequence relation among 3D image blocks should be defined explicitly. Two 3D image blocks are defined as adjacent blocks in a sequence if they satisfy the following conditions: (1) The distance between their anchors is less than or equal to 100 voxels. (2) The node distance between their corresponding nodes on the gold standard reconstruction is 100 nodes. Starting from any 3D image block and according to the above defined adjacency relation, many block sequences with different lengths can be obtained from our 3D image blocks.

**Fig. 5:**
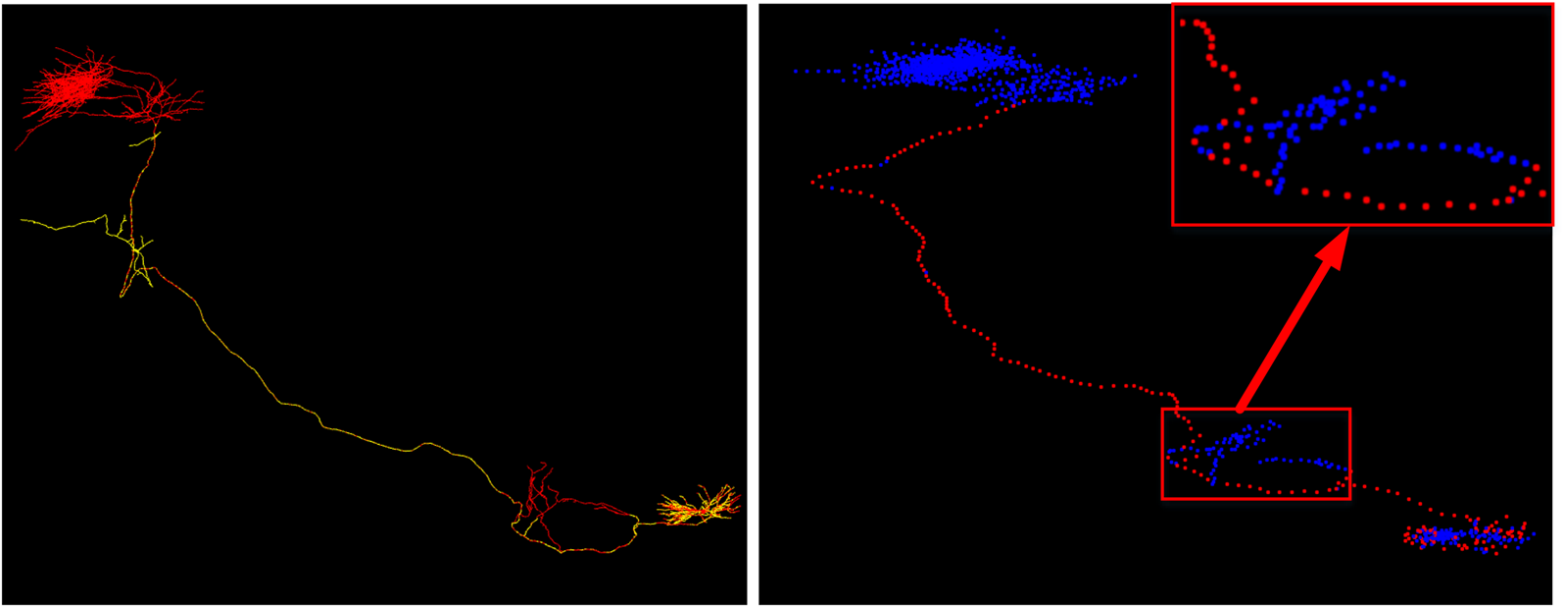
The gold standard reconstruction and APP2 reconstruction of a neuron (left), locations of low-TDBs and high-TDBs on the neuron (right)

Intuitively, most adjacent blocks have the same tracing difficulty. As shown in Fig. 5, the red line and the yellow line in the left picture are the gold standard reconstruction and APP2 reconstruction of a neuron, and red dots and blue dots in the right picture represent locations of low-TDBs and high-TDBs, respectively. From Fig. 5, it can be seen that most red dots or blue dots are continuously scattered on the gold standard line. That is to say, adjacent 3D image blocks have the same tracing difficulty. Furthermore, this observation is tested on 18074 samples from brain-A. For each 3D image block, a block sequence with length 2 is generated, and the probability that two blocks in all 18074 sequences have the same label is 60.55%.

Based on SFE, SIE learns the sequence information hidden in 3D image blocks by two LSTMs, 3D image block based LSTM and auto block based LSTM. Let $X_{I}=\{X_{I1}, X_{I2} \ldots X_{I(n-1)}, X_{In}\}$ be *n* 3D image blocks, $X_{A}=\{X_{A1}, X_{A2} \ldots X_{A(n-1)}, X_{An}\}$ be 32 L-Measure features of their corresponding *n* auto blocks, and $L=\{L_{1}, L_{2} \ldots L_{n-1}, L_{n}\}$ be their one-hot encoding labels. Suppose that $X_{Is}^{\prime }=(X_{I1}^{\prime }, X_{I2}^{\prime } \ldots X_{I(s-1)}^{\prime }, X_{Is}^{\prime })$ and $X_{As}^{\prime }=(X_{A1}^{\prime }, X_{A2}^{\prime } \ldots X_{A(s-1)}^{\prime }, X_{As}^{\prime })$ are two sequences with length *s* constructed from $X_{I}$ and $X_{A}$, and $L_{s}^{\prime }=(L_{1}^{\prime }, L_{2}^{\prime } \ldots L_{s-1}^{\prime }, L_{s}^{\prime })$ be their one-hot encoding labels, where $X_{Ii}^{\prime }\in X_{I}$ and $X_{Ai}^{\prime }\in X_{A}$ ($i=1,2,\cdots , s$). Denote the 3D-ResNet output of $X_{Is}^{\prime }$ by $O_{Is}^{\prime }$and the FCNN output of $X_{As}^{\prime }$ by $O_{As}^{\prime }$, where 3D-ResNet and FCNN are these two neural networks in SFE.

For the 3D image block based LSTM, $L_{s}^{\prime }$ and $O_{Is}^{\prime }$ are concatenated to get sequences $L_{Is}^{\prime }=\{L_{s}^{\prime }, O_{Is}^{ \prime }\}$. Likewise, for the auto block case, $L_{As}^{\prime }=\{L_{s}^{\prime }, O_{As}^{ \prime }\}$ are generated from $L_{s}^{\prime }$ and $O_{As}^{\prime }$. Then, two LSTMs are used to learn $L_{Is}^{\prime }$ and $L_{As}^{\prime }$, and their outputs are denoted by

1 $$\begin{aligned} \begin{aligned}O_{Is}=W_{Is}L_{Is}^{\prime } \ \ \text {and} \ \ O_{As}=W_{As} L_{As}^{\prime }, \end{aligned} \end{aligned}$$

where $W_{Is}$ and $W_{As}$ are learnable parameters, and elements of $O_{Is}$ and $O_{As}$ belong to $\{0,1\}$. In the training stage, optimal values of parameters $W_{Is}$ and $W_{As}$ are obtained, and in the reference stage, outputs $O_{Is}$ and $O_{As}$ can be generated by using these trained parameters.

### MF of 3D-SSM

Model fusion (MF) is used to integrate features and sequence information in 3D image blocks and auto blocks. MF is composed of a concatenation operation and a FCNN to fuse the output features of SIE. The FCNN has two linear layers, and the number of nodes in the first layer and the second layer are 30 and 2, respectively. MF can be expressed by the following formula:

2 $$\begin{aligned} \begin{aligned}O_{M}= softmax \left( W_{M} * cat \left( O_{Is}, O_{As}\right) \right) , \end{aligned} \end{aligned}$$

where *cat* is a concatenation operation, $W_M$ is learnable parameters of FCNN, and $O_M$ is the output of MF.

## Experiment

In this section, we validate the performance of the automatic labeling algorithm and each module of the proposed 3D-SSM model. Furthermore, modules of 3D-SSM trained on samples from brain-A are used to classify samples from brain-B.

### Experimental setup

Our experimental data includes 18074 3D image blocks from brain-A and 5109 3D image blocks from brain-B, and their corresponding gold blocks and auto blocks. We extract 7 neuron distance features, 3 neuronal morphology features and 32 L-Measure features from gold blocks and auto blocks, and manually label 2954 3D image blocks from brain-A as low-TDB or high-TDB. Using these data and with the help of publicly available packages Pytorch [31] and Scikit-learn [32], several algorithms for automatic labeling and three modules of 3D-SSM are implemented on two NVIDIA P5000 GPUs with 16GB memory. We run all algorithms five times and report their average accuracy rates and F1 scores with corresponding standard deviations.

**Table 2:** Results of automatic labeling by FCNN, LR and SVM

Dataset	FCNN		LR		SVM	
	Accuracy	F1	Accuracy	F1	Accuracy	F1
Training	$\underline{96.92\pm 0.19}$	$\underline{96.86\pm 0.20}$	96.05±0.26	95.98±0.27	96.50±0.22	96.44±0.23
Test	$\underline{96.49\pm 0.59}$	$\underline{96.40\pm 0.61}$	96.07±0.53	95.98±0.54	96.30±0.57	96.22±0.59

Numbers with underline are the best results among all models

### Results of automatic labeling

2954 pairs of a gold block and an auto block with manual label are randomly divided into 70% (2068) training samples and 30% (886) test samples. 7 neuron distance features and 3 neuronal morphology features are used to describe the similarity between each pair of gold block and its corresponding auto block. According to the similarity of each pair, their corresponding 3D image block is labeled as low-TDB or high-TDB. We implement FCNN, logistic regression (LR) and Support Vector Machines (SVM) to label a 3D image block, and their average accuracy rates and F1 scores (standard deviation) are given in Table 2.

From Table 2, it can be seen that all three algorithms have quite good performance (about 96% accuracy rates and F1 scores) on both training and test sets. Among them, FCNN generates the best training and test results and is selected to label 3D image blocks without manual labels. For 15120 (18074−2954) 3D image blocks from brain-A, FCNN labels 8423 blocks as low-TDB and 6697 blocks as high-TDB. And for 5109 3D image blocks from brain-B, it labels 3416 blocks as low-TDB and 1693 blocks as high-TDB.

### Results of the SFE module

We take 5342 (29.55%) samples from 23 neurons of brain-A as test set, and 12372 samples from other 70 (93−23) neurons of the same brain as training set. For 3D image block based tracing difficulty classification, the training set is enhanced by rotating 3D images with 90, 180 and 270 degrees along the X-axis direction, and 37116 new training samples are obtained.

Usual 2D Resnet [12], MobileV2 [33], DenseNet [34] and SENet [35] are extended to three dimensions (3D) and implemented to classify the tracing difficulty of 3D image blocks. Adam optimizer [36] with $\beta _1=0.9$ and $\beta _2=0.99$ is applied to optimize these models, and their batch size, initial learning rate and weight decay are set to 30, 0.001 and 0.01, respectively. As given in Table 3, 3D-ResNet achieves the best test accuracy rate, so it is selected as a base method in the 3D-SSM model and its trained parameters are saved for the training of 3D-SSM.

**Table 3:** Feature maps of 3D image blocks based classification results by 3D-ResNet, 3D-MobileNetV2, 3D-DenseNet and 3D-SENet

Dataset	3D-ResNet		3D-MobileNetV2		3D-DenseNet		3D-SENet	
	Accuracy	F1	Accuracy	F1	Accuracy	F1	Accuracy	F1
Training	83.50±0.14	85.18±0.13	79.97±0.18	81.89±0.16	$\underline{84.69\pm 0.16}$	$\underline{86.33\pm 0.14}$	82.29±0.05	84.16±0.04
Test	$\underline{81.29\pm 0.39}$	$\underline{82.78\pm 0.61}$	78.40±0.23	79.83±0.22	72.03±2.47	75.50±1.29	80.11±0.50	81.63±0.92

Numbers with underline are the best results among all models

**Table 4:** 32 L-Measure features of auto blocks based classification results by FCNN, LR and SVM

Dataset	FCNN		LR		SVM	
	Accuracy	F1	Accuracy	F1	Accuracy	F1
Training	$\underline{78.03\pm 0.14}$	$\underline{77.71\pm 0.18}$	75.38±0.20	75.24±0.20	73.54±0.14	73.55±0.14
Test	$\underline{77.97\pm 0.34}$	$\underline{77.63\pm 0.31}$	75.34±0.26	75.17±0.25	73.28±0.35	73.27±0.35

Numbers with underline are the best results among all models

**Fig. 6:**
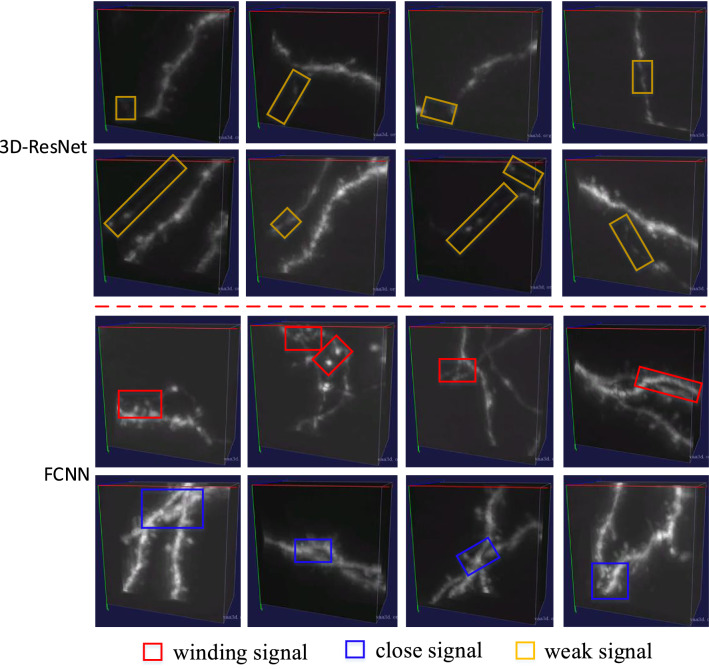
Examples of three types of misclassified samples by the 3D image block based 3D-ResNet and the auto block based FCNN

For auto block based tracing difficulty classification, 32 L-Measure features of auto blocks are used as the input of an algorithm to generate the label of corresponding 3D image blocks. FCNN, LR and SVM are implemented on these features of training set and test set, and their results are given in Table 4. From Table 4, it can be seen that FCNN has the best performance, and so it is adopted as the algorithm for auto blocks based tracing difficulty classification and its parameters are saved for the training of the 3D-SSM model.

**Fig. 7:**
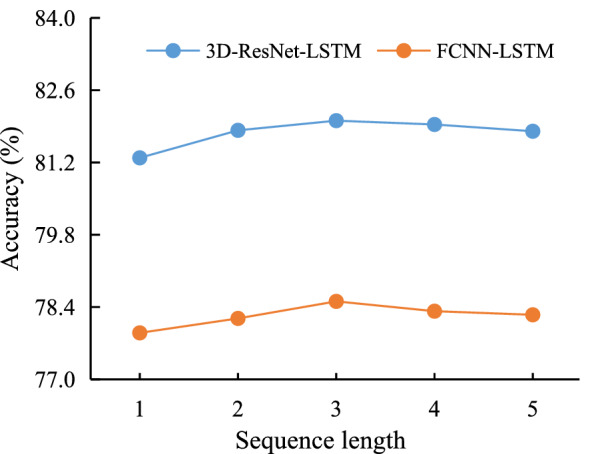
Accuracy rates of 3D-ResNet-LSTM and FCNN-LSTM with different sequence lengths

From Table 3 and Table 4, we can see that the 3D image block based 3D-ResNet has better performance than the auto block based FCNN. Since 3D-ResNet and FCNN utilize feature map of images and morphology features of reconstructions respectively, labels generated by them might be quite different. We visually check their misclassified samples in the test set, and summarize three main types of errors. As illustrated in Fig. 6, 3D-ResNet does badly on 3D image blocks with simple and weak signals (yellow blocks in Fig. 6), while FCNN mainly makes mistakes on 3D image blocks with winding signals (red blocks in Fig. 6) or close signals (blue blocks in Fig. 6) from different neuronal segments. The possible reason is that 3D-ResNet pays more attention to the overall structure of neuronal signals and FCNN focuses on the complexity of the geometry of neuronal signals. Hence, the fusion of the 3D image block based 3D-ResNet and the auto block based FCNN would provide more discriminative features for the classification task, which will be discussed in subsection 4.5.

### Results of the SIE module

Two LSTMs are utilized to learn sequence information hidden in 3D image blocks, one is for the output of the 3D image block based 3D-ResNet (3D-ResNet-LSTM) and the other is for the auto block based FCNN (FCNN-LSTM). Test accuracy rates of 3D-ResNet-LSTM and FCNN-LSTM with sequence length varying from 1 to 5 are given in Fig. 7. From Fig. 7, it can be seen that LSTMs with sequence length 3 can improve accuracy rates of 3D-ResNet and FCNN about 0.8 and 0.5 percentage points, respectively. If the sequence length is larger than 3, 3D image blocks in a sequence span a large area. In this case, the complexity of neuronal morphology structures and the signal-to-noise ratio (SNR) of signals might change greatly. So the tracing difficulty of different 3D image blocks in the sequence might be different (low or high). This explanation is verified by the downward trend of curves in Fig. 7.

**Table 5:** Results of the 3D-SSM model with different sequence lengths in LSTMs

Dataset	Length = 2		Length = 3		Length = 4		Length = 5	
	Accuracy	F1	Accuracy	F1	Accuracy	F1	Accuracy	F1
Training	87.02±0.16	88.46±0.13	$\underline{87.04\pm 0.05}$	$\underline{88.50\pm 0.04}$	86.99±0.14	88.24±0.14	86.96±0.16	88.22±0.15
Test	83.87±0.09	85.43±0.10	$\underline{84.07\pm 0.17}$	$\underline{85.47\pm 0.28}$	83.63±0.13	85.42±0.15	83.60±0.15	85.40±0.18

Numbers with underline are the best results among all models

**Fig. 8:**
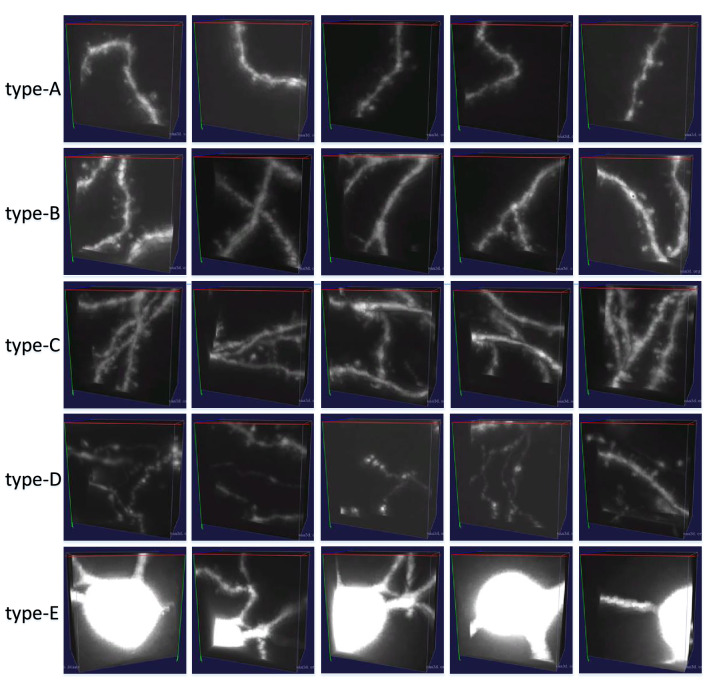
Examples of five types of correctly classified samples by 3D-SSM

### Results of the 3D-SSM model on brain-A

The trained 3D-ResNet and FCNN in SFE, and two trained LSTMs in SIE are used to construct the 3D-SSM model (model fusion), and their trained parameters are adopted to train parameters in the FCNN of MF. Table 5 gives accuracy rates of the 3D-SSM model with different sequence lengths in LSTMs. Its best results are 87.04% and 84.07% on the training set and test set, respectively.

We visually check all correctly classified test samples by 3D-SSM, and summarize them into five main types: type-A, type-B, type-C, type-D and type-E. Type-A are 3D image blocks with only one neuronal segment and without weak signal, type-B are blocks with a few neuronal segments and a high SNR, type-C are blocks with much more neuronal segments and interlaced neuronal signals, type-D are blocks with many weak neuronal signals and complex neuronal morphologies, and type-E are blocks with a soma. The former two types belong to low-TDB, the latter three types are high-TDB, and type-C and type-D compose the majority of the high-TDB category. Five examples of each type are demonstrated in Fig. 8. It can be seen that the 3D-SSM model correctly classifies 3D image blocks with different directions, lengths, SNRs, numbers of bifurcations and numbers of neuronal segments.

### Results of the 3D-SSM model on brain-B

In order to verify the generalization of SEF, SIF and the 3D-SSM model, we use the best parameters (among five runs) trained on samples from brain-A to classify samples from brain-B. Table 6 gives results of the trained 3D-ResNet-LSTM, FCNN-LSTM and 3D-SSM mode on samples from brain-B. Comparing Fig. 7, Table 5 and Table 6, it can be seen that 3D-ResNet-LSTM has the same performance on brain-B, FCNN-LSTM has little worse performance on brain-B, and 3D-SSM even has better F1 score on this brain. While the sequence length equals 3, 3D-SSM produces an accuracy rate 83.21%. These experimental results show that 3D-SSM has good performance on classifying 3D image blocks from non-training whole mouse brains.

**Table 6:** Classification results of trained models with different sequence lengths on brain-B

Length	3D-ResNet-LSTM		FCNN-LSTM		3D-SSM	
	Accuracy	F1	Accuracy	F1	Accuracy	F1
2	82.32	86.83	75.20	78.96	82.31	86.53
3	82.17	86.67	74.59	78.29	83.21	87.26
4	82.07	86.61	74.85	78.55	82.91	86.97
5	82.46	87.01	73.75	77.43	83.03	87.14

Numbers with underline are the best results among all models

## Conclusions and discussions

In this paper, we construct 3D image blocks, gold blocks, and auto blocks from 3D neuron images of two whole mouse brains, and extract 7 neuron distance features, 3 neuronal morphology features and 32 L-Measure features from gold blocks and auto blocks. 3D image blocks are labeled by manual or a FCNN, and the sequence relation among them is built. More importantly, a 3D-SSM model is proposed to classify the tracing difficulty of 3D image blocks, which has three modules: SEF, SIE and MF. SEF consists of a 3D-ResNet and a FCNN for extracting structure features of 3D image blocks and auto blocks. SIE adopts two LSTMs to extract sequence information hidden in 3D image blocks, and MF fuses different features in SIE. These modules are validated on more than 20000 samples from two whole mouse brains. In addition, three types of misclassified samples by SFE and five types of correctly classified samples by 3D-SSM are summarized. Classification results on the tracing difficulty of 3D image blocks by 3D-SSM can be used as a stop condition for an automatic tracing algorithm in the Ultra-Tracer framework, which is an important factor to realize the interaction between automatic tracing and manual reconstructing.

Although the proposed 3D-SSM model performs well on classifying the tracing difficulty of 3D image blocks, it is still hard to correctly classify 3D image blocks with weak signals, close signals, wind signals, complex neuronal structures and so on. In fact, the tracing difficulty classification task on 3D image blocks is much more difficult than traditional image classification tasks. Firstly, 3D image blocks have a much higher dimension, more parameters need to be learned, and model optimization is more difficult. Secondly, the number of neuronal fragments, signal strengths, signal quantity, signal directions, signal shapes and so on are quite different for different 3D image blocks, so it is hard for a model to learn enough advanced features which can well distinguish low-TDBs and high-TDBs. Although it is difficult to classify the tracing difficulty of 3D image blocks, this task is important for interacting between automatic tracing and manual reconstructing, and we will further explore it from the perspective of producing more accurate data and designing better models.

## Data Availability

The datasets used and/or analyzed during the current study are available from the Southeast University-Allen Institute Joint Center on reasonable request. The codes of models are available at https://github.com/BingooYang/Tracing-difficulty-classification-on-3D-neuron-image-block .
